# Cases of Airy Tumours of the Uterus

**Published:** 1831

**Authors:** 


XXXVIII.

CASES OF AIRY TUMOURS OF THE UTERUS.

IN the Opusculi della Societa Medico-Chirurgica di Bologna, Vol. IV. are two cases of gazeous enlargements of the uterus, which we shall here abridge.

*Case* 1. A plethoric female, in the prime of life, being suddenly exposed to cold during menstruation, the catamenia were suppressed, and an acute pain was soon afterwards felt in the region of the uterus, as well as an enlargement of that organ, which could be felt above the pubes, with pain in the groins. These phenomena were ushered in by a rigor, succeeded by febrile re-action, heat, thirst, &c. On examination, the uterus was found, by percussion, to emit a hollow sound. The female was bled largely from the feet, while emollient fomentations, lavements, &c. were employed. Nevertheless the volume of the uterus enlarged, and became more globular. Leeches were then applied to the pudendum without any advantage. The symptoms augmented in intensity, and the uterus was again examined per vaginam and above the pubes. The finger being introduced into the os uteri an explosion of gazeous fluid took place of fetid odour. The size of the abdomen was quickly diminished; but soon afterwards regained its former dimensions. A tube was then introduced into the womb, giving exit to an immense quantity of gas, with some clots of blood. This state continued some days, when all the phenomena subsided and disappeared.

GAZEOUS COLLECTION IMITATING PREGNANCY.

*Case* 2. A female, 40 years of age, and who never had borne children, began to present signs of pregnancy. The menses which had hitherto been very regular, now stopped, and the os uteri, on examination, was found to be firmly closed. The patient experienced none of the sickness on other usual inconveniences of pregnancy. Nevertheless the uterus, towards the fifth month, reached the umbilicus, and was circumscribed. Towards the end of the sixth month all hopes of pregnancy vanished by the explosion of a large collection of gas from the uterus, upon which the swelling of the abdomen disappeared, and the patient regained her natural state of health.

